# Wooden‐Tip Electrospray Ionization Mass Spectrometry Combined With Machine Learning for Differentiating Thyroid Tumours

**DOI:** 10.1002/ansa.70083

**Published:** 2026-04-11

**Authors:** Da‐Sheng Liu, Li Liu, Baixue Wang, Jianfeng Zhang, Hong‐Guo Lin, Xiang‐Xiong Huang, Kang‐Jian Deng, Yu‐Teng Zhou, Yunlong Pan, Bin Hu, Xue‐Yang Huang

**Affiliations:** ^1^ Department of General Surgery The First Affiliated Hospital of Jinan University Guangzhou China; ^2^ Department of Vascular Thyroid Surgery The Second Affiliated Hospital of Guangzhou University of Chinese Medicine Guangzhou China; ^3^ Thyroid Diagnosis and Treatment Center Guangdong Provincial Hospital of Chinese Medicine Guangzhou China; ^4^ Health Management Center The First Affiliated Hospital of Jinan University Guangzhou China; ^5^ College of Environment and Climate Institute of Mass Spectrometry and Atmospheric Environment Jinan University Guangzhou China

**Keywords:** electrospray ionization, machine learning, mass spectrometry, thyroid cancer, tissue analysis

## Abstract

Direct mass spectrometry (MS) analysis of human tissues at the molecular level has great potential for clinical diagnosis and biomarker discovery. However, conventional MS‐based analytical methods often require complicated and time‐consuming sample preparation, which limits their applicability in rapid clinical analysis. In this study, we developed a rapid analytical strategy by integrating ambient ionization MS with machine learning (ML) for the differentiation of different thyroid tumours. A disposable slim wooden tip (WT) was employed as both a sample holder and an electrospray emitter, enabling direct extraction and ionization of metabolites from tiny thyroid tissue samples under electrospray ionization (ESI) conditions. Using this WT–ESI–MS method, lipid profiles of thyroid tissues could be obtained within minutes without extensive sample preparation. A total of 45 thyroid samples, including 15 healthy tissues, 15 benign tumours and 15 malignant tumours, were analysed. The acquired MS data were further processed using ML‐based classification models to distinguish different tumours and identify potential lipid biomarkers. Structural characterization of representative lipids was also performed by MS/MS analysis. The results demonstrated that this WT–ESI–MS combined with ML provides a rapid and effective approach not only for differentiating tumour tissues and healthy samples but also for benign and malignant tumours, highlighting its potential application in clinical diagnosis and intraoperative tissue evaluation.

## 1 Introduction

Thyroid cancer is one of the most common endocrine malignancies worldwide, and its incidence has increased rapidly over the past decades [1, 2, 3]. Early and accurate diagnosis is essential for guiding clinical treatment and improving patient prognosis. However, distinguishing cancer thyroid tumours from benign thyroid nodules remains a major diagnostic challenge because benign lesions often exhibit clinical and morphological characteristics similar to cancer tumours [4]. Therefore, accurate differentiation among benign tumours, cancer tumours and normal thyroid tissues is crucial for clinical decision‐making.

Various imaging techniques such as ultrasound, computed tomography, magnetic resonance imaging and positron emission tomography are widely used for thyroid disease evaluation [5, 6, 7, 8, 9]. However, they mainly provide anatomical and structural information and lack molecular‐level insights. Therefore, definitive diagnosis still relies on histopathological examination. Conventional pathological workflows require labour‐intensive steps such as fixation, sectioning and staining, which are time‐consuming and may delay molecular analysis. Consequently, molecular analysis of human tissue has become an important strategy in biomedical research and analytical science [6, 7]. Metabolic profiling provides valuable information for biomarker discovery and disease diagnosis and may help distinguish benign and cancer thyroid lesions [10, 11]. Therefore, the development of rapid and reliable analytical techniques for thyroid tissue characterization is of considerable clinical interest.

Currently, advanced techniques such as chromatography, mass spectrometry (MS), and nuclear magnetic resonance are widely used for metabolic analysis and have been applied to metabolite studies of thyroid cancer [5]. Among these methods, MS has emerged as a powerful tool for molecular profiling of biological tissues due to its high sensitivity and broad chemical coverage [12, 13, 14, 15]. However, conventional MS‐based methods typically require complicated sample pretreatment and chromatographic separation prior to analysis [16, 17, 18], which limits their application in rapid analysis of clinical tissue. The development of ambient ionization MS has enabled rapid molecular analysis by allowing direct analysis of complex samples with minimal or no sample preparation [19, 20, 21, 22]. Among ambient ionization techniques, wooden‐tip electrospray ionization mass spectrometry (WT–ESI–MS) has attracted attention as a simple and cost‐effective approach for direct tissue analysis. In WT–ESI–MS, a disposable wooden tip serves simultaneously as a sampling probe and an electrospray emitter, enabling rapid extraction and ionization of analytes directly from tissue surfaces [23, 24]. This technique has been successfully applied to rapid lipid and metabolite profiling in biological samples.

Recently, the integration of MS with artificial intelligence, particularly machine learning, has created new opportunities for biomedical research and disease diagnosis [25, 26, 27, 28, 29, 30]. Multivariate statistical methods such as PCA and partial least squares discriminant analysis (PLS‐DA) are widely used to analyse complex MS datasets and identify discriminative molecular features. Machine learning algorithms such as random forest (RF) can further improve classification performance by enabling nonlinear modelling and feature importance analysis, facilitating the discovery of disease‐related biomarkers [31]. Despite these advances, many previous studies mainly focused on distinguishing cancer tissues from normal tissues, which represents a relatively simplified classification problem. In clinical practice, however, differentiating benign thyroid nodules from cancer tumours is a challenging task because benign lesions often share partially overlapping molecular characteristics with cancer tissues.

In our previous work, WT–ESI–MS was applied to direct analysis of thyroid tissue, enabling discrimination between normal and cancer tissues using multivariate statistical analysis [32]. Although our previous study demonstrated the feasibility of WT–ESI–MS for rapid thyroid tissue characterization, it was limited to binary classification and did not address the challenge of distinguishing benign from cancer thyroid tissues. Here, we have further extended this approach to a clinically relevant three‐class classification of normal, benign and cancer thyroid tissues. Lipid profiles obtained by WT–ESI–MS were analysed using multivariate statistical methods and machine learning algorithms. In particular, an RF model was applied to improve classification robustness and identify discriminative lipid features. By integrating rapid ambient MS with machine learning‐based analysis, this study provides a more comprehensive strategy for thyroid tissue discrimination and highlights the potential of WT–ESI–MS for rapid clinical diagnostics.

## 2 Materials and Methods

### 2.1 Chemicals and Samples

Wooden tips were prepared from natural wooden toothpicks purchased from a local supermarket. Before use, the wooden tips were washed with a methanol solution (methanol/water, 1:1, vol/vol) to remove surface contaminants. Methanol was purchased from Sigma‐Aldrich (St. Louis, USA). Ultrapure water was generated using a Milli‐Q water purification system. Thyroid tissue samples (approximately 1.0 mm × 1.0 mm × 1.0 mm) were collected from patients hospitalized at the Second Affiliated Hospital of Guangzhou University of Chinese Medicine (Guangzhou, China). Written informed consent was obtained from all patients prior to sample collection. To ensure good stability, each wooden stick was manufactured with a consistent tip size (0.1 mm) and underwent inspection and quality control under a microscope.

This study was approved (approval number: ZE2022‐371) by the Ethics Committee of the Second Affiliated Hospital of Guangzhou University of Chinese Medicine (Guangzhou, China). Routine histopathological diagnosis of thyroid tissues was performed using paraffin‐embedded tissue sections [32].

### 2.2 Wooden‐Tip ESI–MS Analysis

**FIGURE 1:**
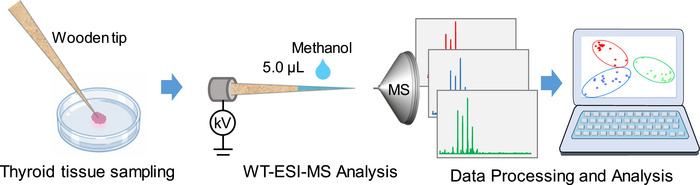
Schematic diagram of the analytical workflow for WT–ESI–MS analysis of thyroid tissue samples.

All MS experiments were carried out on a high‐resolution Orbitrap‐QE mass spectrometer (Thermo Fisher Scientific, Bremen, Germany). The fabrication and application of wooden‐tip ESI–MS were performed according to our previous study [32]. Briefly, the wooden tip was cut into a small probe (∼ 1.0 mm), as shown in Figure 1. The wooden tip was first pre‐wetted with pure water and then gently contacted with the thyroid tissue for touch sampling with a sampling depth of approximately 1.0 mm for 10 s. After sampling, the wooden tip was mounted onto a commercial nanoESI device (Thermo Fisher Scientific, Bremen, Germany). The distance between the wooden tip end and the MS inlet was approximately 1.0 cm in the horizontal direction. Subsequently, 5.0 µL of pure methanol was added onto the wooden tip to extract analytes, and a high voltage of 3.5 kV was applied to generate spray ionization for MS analysis. Pure methanol was used not only to efficiently extract lipids but also to increase the mass spectral response by increasing solvent volatility and decreasing surface tension on wooden tips [13, 33]. The ionization voltage of 3.5 kV was used in wooden tip, which is higher than the normal capillary ESI voltage (3.0 kV), because the tip‐end size of wooden tip (0.1 mm) is larger than ESI capillary.

For MS/MS experiments under CID mode, precursor ions were isolated with a window of 0.4 Da and fragmented with a collision energy of 40%. All MS data acquisition and processing were performed using Xcalibur software (Thermo Fisher Scientific, Bremen, Germany). The acquisition speed was 5.0 scans/s. Typically, mass spectra acquired over 1 min were averaged to obtain the final high‐resolution MS spectra. Each sample (*n* = 45) was analysed three times. For each analysis, a new wooden tip was used to independently perform tissue sampling and spray ionization. Therefore, three independent MS datasets were obtained for each sample, resulting in a total of 135 MS datasets for subsequent machine learning analysis.

### 2.3 Data Analysis With ML Approaches

MS peaks within the *m/z* range of 400–2000 were extracted from each spectrum and normalized prior to statistical analysis. Only peaks with relative abundances ≥ 1.0% were retained and used as input variables for subsequent analysis. PLS‐DA and RF were applied to investigate the differences among thyroid tissue types, as described in previous studies [26, 34]. PLS‐DA was performed using SIMCA 14 software (Umetrics, Sweden) to visualize clustering patterns and evaluate the discrimination among different tissue groups. RF analysis was implemented in Python (version 3.10.0) using the scikit‐learn library. For model development, the dataset was randomly divided into a training set (70%) and a test set (30%), following the previous optimal ratio for data splitting [35, 36]. The training set was used to construct the RF classification model, and the test set was used to evaluate the predictive performance of the model. The hyperparameters of the RF model were optimized using the GridSearchCV function implemented in the scikit‐learn library.

## 3 Results and Discussion

### 3.1 Lipid Profiles of Different Thyroid Tissues

**FIGURE 2:**
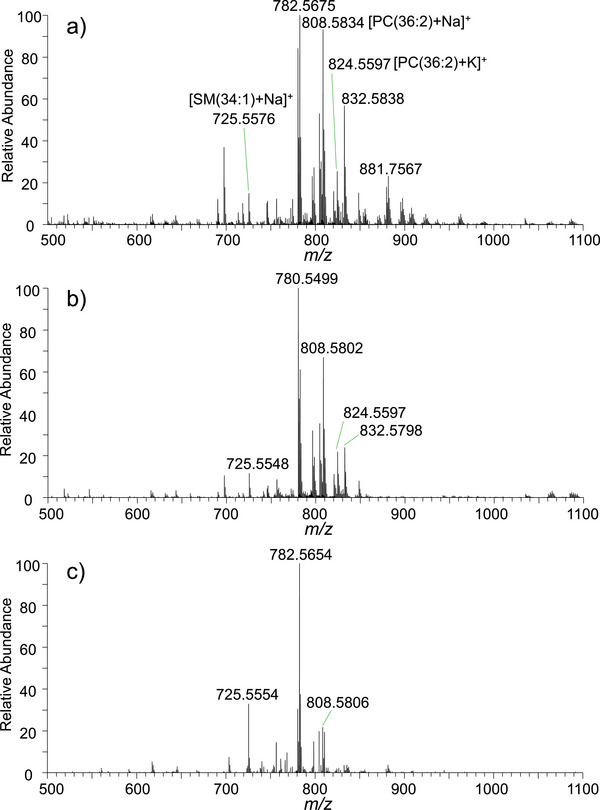
Typical lipid profiles obtained by WT–ESI–MS analysis of three types of thyroid tissue samples: (a) normal tissue, (b) benign tissue and (c) cancer tissue.

Figure 2a shows a representative high‐resolution mass spectrum obtained from normal thyroid tissue using WT–ESI–MS. Numerous MS peaks were detected across the acquired mass range, with the most intense signals concentrated between *m/z* 700 and 1000. For clarity, only the representative *m/z* range of 500–1100 is displayed in Figure 2. Several characteristic peaks, including *m/z* 725.5554, 782.5675, 808.5834 and 832.5838, were observed with relatively high intensities. Because direct MS analysis of raw tissue samples without LC separation, absolute intensity and dateable peak of lipids may be affected due to the ion suppression. These ions are tentatively attributed to lipid species, indicating that lipid molecules were efficiently extracted from thyroid tissue using the WT‐ESI sampling approach. Therefore, without LC separation, the abundant lipid profile could show the characteristic of thyroid tissue. To evaluate the reproducibility, these lipid ions were also detected in six parallel experiments of same healthy tissue with different wooden tips (Table S1), showing a well‐acceptable relative standard deviation (RSD) ranging from 5.3% (*n* = 6) to 9.8% (*n* = 6). Similar lipid‐related peaks were also detected in benign thyroid tissue (Figure 2b). However, the relative intensity patterns of several characteristic ions differed from those obtained in normal tissue. For instance, variations in signals at *m/z* 780.5499, 782.5675 and 808.5802 were observed, suggesting differences in lipid composition between normal and benign thyroid tissues. In contrast, the mass spectrum obtained from thyroid cancer tissue (Figure 2c) exhibited a distinct spectral pattern compared with non‐cancer tissues. The peak at *m/z* 782.5654 became the dominant signal. Several other lipid‐related peaks showed relatively lower intensities. These spectral differences suggest that lipid metabolism in thyroid tissue undergoes noticeable alterations during tumour development.

Compared with healthy thyroid tissue, the observed changes in lipid signals in benign and cancer tissues are likely associated with tumour‐related metabolic alterations. The distinct lipid profiles among normal, benign and cancer thyroid tissues indicate that high‐resolution MS can capture molecular differences that are potentially useful for tissue differentiation. Many lipids have been reported as potential biomarkers in thyroid cancer [37, 38]. Alterations in lipid metabolism have recently attracted increasing attention in cancer research, as tumour cells often exhibit enhanced fatty acid synthesis and turnover to support rapid proliferation and membrane biosynthesis [39]. Several lipid metabolic pathways have also been proposed to discriminate between normal and cancer cells [40]. However, most lipidomic studies of thyroid tissues have been performed using conventional MS approaches that require complicated sample preparation and chromatographic separation. In contrast, the ambient MS strategy employed in this work enables rapid detection of thyroid lipids within minutes, providing a potential advantage for rapid molecular assessment of thyroid tissue. Overall, the lipid profile differences observed in the WT–ESI–MS spectra provide important molecular information for distinguishing thyroid tissue types and form the basis for subsequent multivariate statistical and machine learning analyses.

### 3.2 Identification of Typical Lipids

**FIGURE 3:**
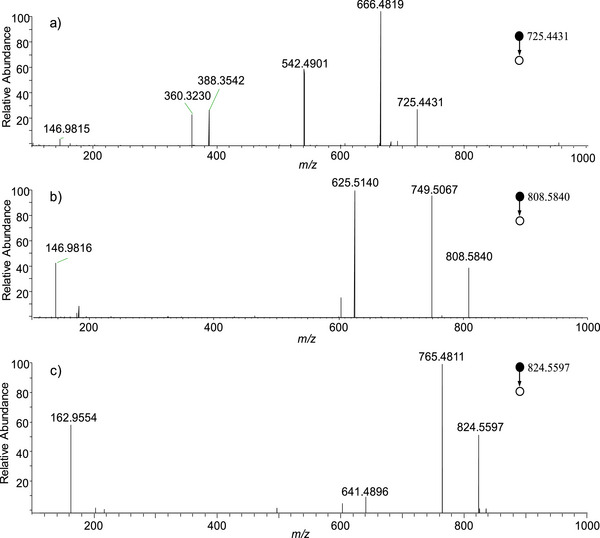
MS/MS spectra of typical lipid adduct ions detected in thyroid tissues: (a) sodium adduct of SM(34:1), (b) sodium adduct of PC(36:2) and (c) potassium adduct of PC(36:2).

To further characterize the major lipid species detected in thyroid tissues, several representative high‐intensity ions were selected for MS/MS analysis. In particular, the precursor ions at *m/z* 725.4431, 808.5840 and 824.5597 were chosen because they were consistently observed with relatively high intensities in the mass spectra and represent typical lipid signals detected in thyroid tissue samples. The high‐resolution MS/MS spectra of these precursor ions are shown in Figure 3. For the precursor ion at *m/z* 725.4431, several characteristic fragment ions were observed at *m/z* 146.9815, 360.3230, 388.3542, 542.4901 and 666.4819 (Figure 3a). Based on the accurate mass measurement and comparison with previously reported MS/MS data, this ion was tentatively assigned as the sodium adduct of sphingomyelin, [SM(34:1)+Na]^+^ [41]. Sphingomyelin is a major component of eukaryotic cell membranes and plays an important role in membrane structure and cellular signalling [37, 42]. Recent studies have suggested that sphingomyelin is also involved in the regulation of cellular physiological processes in thyroid tissues [42].

For the precursor ion at *m/z* 808.5840 (Figure 3b), the MS/MS spectrum exhibited characteristic fragment ions associated with the phosphatidylcholine (PC) head group. In particular, the abundant product ion at *m/z* 749.5067 corresponds to the neutral loss of the choline moiety (N(CH_3_)_3_) from the precursor ion. In addition, the fragment ion at *m/z* 146.9815 corresponds to the sodium adduct of the phosphocholine fragment ([HPO_4_CH_2_CH_2_+Na]^+^), which is a well‐known diagnostic ion for PC lipids in positive ion mode. Based on the accurate mass and characteristic fragment ions, this precursor ion was assigned as [PC(36:2)+Na]^+^. Similarly, the precursor ion at *m/z* 824.5597 produced a characteristic fragment ion at *m/z* 162.9554 (Figure 3c), corresponding to the potassium adduct of the phosphocholine fragment ([HPO_4_CH_2_CH_2_+K]^+^). Combined with the accurate mass measurement, this ion was assigned as the potassium adduct of PC, [PC(36:2)+K]^+^. Furthermore, more lipid ions such as [PC(16:0/18:1)+K]^+^, [PC(16:0/18:1)+Na]^+^, [PC(16:0/18:2)+Na]^+^ and [PC(14:0/18:0)+Na]^+^ were identified by MS/MS experiment (Figure S2). It should be noted that fragment ions derived from the phosphocholine head group are commonly observed diagnostic ions for PC lipids. The proposed lipid identifications were supported by accurate mass measurements obtained from high‐resolution MS, characteristic MS/MS fragment ions and comparison with previously reported literature and lipid databases such as LIPID MAPS. Although the relative abundances of fragment ions may vary due to different adduct forms or experimental conditions, these characteristic fragments provide reliable evidence for the tentative identification of PC species. These results provide structural evidence for the major lipid species detected in thyroid tissues and support subsequent lipid biomarker screening. The identified lipid species are consistent with previously reported lipid alterations in thyroid tissues associated with tumour development and may serve as potential biomarkers related to the biological behaviour and metabolic alterations of thyroid cancer [32, 43, 44].

### 3.3 Biomarker Discovery

**FIGURE 4:**
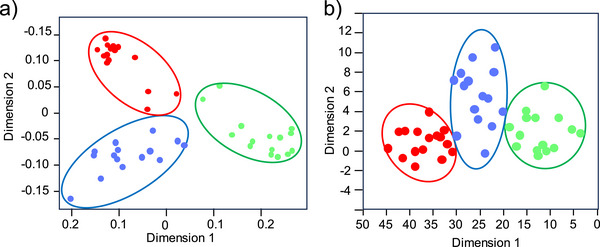
Multivariate analysis of MS data obtained from three types of thyroid tissue samples: (a) MDS plot based on RF analysis and (b) PLS‐DA score plot. Red: cancer tissue; green: normal tissue; blue: benign tissue.

In this study, an RF algorithm based on a decision tree strategy was employed to identify the most important lipid variables that discriminate different thyroid tissue samples. RF is widely used in metabolomics studies because of its strong classification capability and robustness in handling complex datasets with multiple variables. Figure 4a shows the multidimensional scaling (MDS) plot generated from the RF model, which classifies lipid metabolites detected from healthy, benign and cancer thyroid tissue samples. It can be observed that the lipid metabolite profiles from the three groups form distinct clusters, showing that significant lipid differences exist among normal, benign and cancer thyroid tissues. To further evaluate the predictive performance of the RF classifier, the dataset was randomly divided into training and testing sets. In this procedure, 70% of the samples were used as the training set to construct the classification model, and the remaining 30% were used as the test set for model validation. Receiver operating characteristic (ROC) analysis was then performed to assess the diagnostic performance of the RF model. The area under the curve (AUC) values were 0.9785, 0.9699 and 0.9798 for healthy, benign and cancer samples, respectively (Figure S1). These high AUC values indicate that the RF model possesses excellent classification ability for distinguishing the three types of thyroid tissues based on their lipid profiles.

In addition to the RF classification model, multivariate statistical analysis was performed to further investigate lipid metabolic differences among the three groups. The PLS‐DA score plot (Figure 4b) shows a clear separation among the healthy, benign and cancer groups, indicating that the lipids of different thyroid tissues are significantly different. To further identify potential lipid biomarkers responsible for the observed metabolic differences, the variable importance in projection (VIP) scores derived from the PLS‐DA model were used to rank the contribution of each lipid species to group discrimination. In this study, metabolites with VIP scores greater than 1.0 were considered potential biomarker candidates, whereas those with VIP scores greater than 1.2 were regarded as the most significant biomarkers contributing to the differentiation of the three groups. All lipid species meeting this threshold (VIP > 1.2) were summarized in Table 1. Interestingly, many of the selected biomarker candidates belong to the PC lipid class. PC metabolism is closely associated with membrane remodelling and cellular signalling processes. During phospholipid metabolism, phospholipase hydrolyses glycerophospholipids to generate lysophosphatidylcholine and fatty acids, which play important roles in cell proliferation, membrane integrity and tumour development. PC and choline metabolites generated during this metabolic process are closely related to the proliferation and survival of tumour tissues [45, 46]. Previous studies have reported that alterations in PC metabolism are closely associated with thyroid cancer progression. For example, plasma PC levels were found to be significantly reduced in patients with thyroid cancer compared with healthy individuals [47]. In addition, Jiang et al. [37] also demonstrated that specific PC species were downregulated in thyroid cancer patients. Despite the ion suppression in direct tissue samples, we maintained consistent sampling and ionization conditions across all samples and applied normalization to reduce variability. Our multivariate models rely on relative pattern (fingerprint profile) recognition rather than absolute intensities, and the strong classification performance suggests that the observed metabolic differences are robust. Overall, the combination of ambient MS‐based lipid profiling with machine learning approaches provides a rapid and effective strategy for distinguishing different thyroid tissue types. Compared with conventional analytical methods used for thyroid cancer diagnosis, this ambient MS‐based method coupled with ML algorithms offers several advantages, including minimal sample preparation, rapid analysis and high accuracy.

**TABLE 1:** Lipid information for variable importance in projection values larger than 1.2.

Ions	Variable importance in projection values	Lipids	Formula	Adducts
782.5669	2.22052	PC(16:0/18:1)	C_42_H_8_2NO_8_P	[M+Na]^+^
809.5853	1.65238	PG(37:0(OH))	C_43_H_85_O_11_P	[M+H]^+^
788.5475	1.61606	PC(O‐36:5)	C_44_H_80_NO_7_P	[M+Na]^+^
781.5535	1.5627	PA(O‐40:2)	C_43_H_83_O_7_P	[M+K]^+^
806.5658	1.54366	PC(18:1/18:2)	C_44_H_82_NO_8_P	[M+Na]^+^
766.5376	1.40638	PC(16:0/17:2)	C_41_H_78_NO_8_P	[M+Na]^+^
820.5243	1.34875	PC(18:2/18:2)	C_44_H_80_NO_8_P	[M+K]^+^
824.5548	1.27232	PC(18:1/18:1)	C_44_H_84_NO_8_P	[M+K]^+^
799.5439	1.26545	PG(36:1)	C_42_H_81_O_10_P	[M+Na]^+^
895.7132	1.25544	TAG(16:0/18:1/18:2)	C_55_H_100_O_6_	[M+K]^+^
810.5971	1.19992	PC(18:0/20:4)	C_46_H_84_NO_8_P	[M+H]^+^
780.5515	1.19127	PC(16:0/18:2)	C_42_H_80_NO_8_P	[M+Na]^+^
805.5544	1.15281	PG(37:2(OH))	C_43_H_81_O_11_P	[M+H]^+^
897.7296	1.14604	TAG(16:0/16:1/20:1)	C_55_H_102_O_6_	[M+K]^+^
798.5393	1.14388	PC(16:0/18:1)	C_42_H_82_NO_8_P	[M+K]^+^
796.5240	1.11898	PC(16:0/18:1)	C_42_H_82_NO_8_P	[M+K]^+^
774.5406	1.11526	PE(O‐38:5)	C_43_H_78_NO_7_P	[M+Na]^+^
833.5884	1.09329	PG(39:2(OH))	C_45_H_85_O_11_P	[M+H]^+^
797.5277	1.08881	PG(38:5)	C_44_H_77_O_10_P	[M+H]^+^
756.5512	1.02268	PC(14:0/18:0)	C_40_H_80_NO_8_P	[M+Na]^+^
773.5289	1.08735	PG(34:0)	C_40_H_79_O_10_P	[M+Na]^+^

## 4 Conclusion

In conclusion, we applied an ambient MS strategy based on wooden‐tip electrospray ionization to investigate lipid profiles of normal, benign and cancer thyroid tissues. The WT–ESI–MS method enables direct analysis of tiny tissue samples using a disposable wooden tip with minimal sample preparation and without chromatographic separation. Under these conditions, molecular information from each tissue sample can be rapidly obtained within minutes. The obtained mass spectra revealed that lipids were the dominant molecular species in thyroid tissues, and the lipid profiles provided important information for distinguishing different types of thyroid tissues. High‐resolution MS combined with MS/MS experiments enabled reliable identification and structural characterization of representative lipid species. In addition, machine learning and multivariate statistical approaches, including RF and PLS‐DA analysis, were applied to the acquired MS data to identify potential lipid biomarkers and improve tissue classification performance. Overall, this study demonstrates that WT–ESI–MS combined with machine learning provides a rapid, simple and reliable analytical strategy for thyroid tissue analysis, highlighting its potential for rapid clinical diagnosis and intraoperative tissue evaluation of thyroid cancer.

## Author Contributions

**Da‐Sheng Liu**: conceptualization, methodology, funding acquisition, project administration, resources, writing – original draft, writing – review and editing. **Li Liu**: conceptualization, investigation, validation, visualization, writing – original draft, writing – review and editing. **Baixue Wang**: methodology, software, data curation, investigation, validation, formal analysis. **Jianfeng Zhang**: methodology, software, data curation, investigation, validation, formal analysis. **Hong‐Guo Lin**: supervision, resources. **Xiang‐Xiong Huang**: supervision, resources. **Kang‐Jian Deng**: supervision, resources. **Yu‐Teng Zhou**: supervision, resources. **Yunlong Pan**: supervision, project administration, resources, writing – original draft, writing – review and editing. **Bin Hu**: conceptualization, supervision, writing – original draft, writing – review and editing. **Xue‐Yang Huang**: supervision, resources, writing – original draft, writing – review and editing. All the authors reviewed this manuscript and approved the submitted version.

## Conflicts of Interest

The authors declare no conflicts of interest.

## Supporting information




**Supporting File**: ansa70083‐sup‐0001‐SuppMat.docx.

## Data Availability

The data that support the findings of this study are available in the Supporting Information of this article.
