# Supplementary material for: Wooden‐Tip Electrospray Ionization Mass Spectrometry Combined With Machine Learning for Differentiating Thyroid Tumours
Source: Anal Sci Adv. 2026 Apr 11;7(1):e70083. doi: 10.1002/ansa.70083 (PMC13069969; doi:10.1002/ansa.70083)
Supplement: Supplementary file 1 — Supporting File: ansa70083‐sup‐0001‐SuppMat.docx. [file ANSA-7-e70083-s001.docx]

*Supplementary material*

**Wooden-Tip Electrospray Ionization Mass Spectrometry Combined with Machine Learning** **for Differentiating Thyroid Tumors**

Da-Sheng Liu^1,2,3#^, Li Liu^4#^, Baixue Wang^5^, Jianfeng Zhang^5^, Hong-Guo Lin^2,3^, Xiang-Xiong Huang^2,3^, Kang-Jian Deng^2,3^, Yu-Teng Zhou^2,3^, Yun-Long Pan^1*^, Bin Hu^5*^, Xue-Yang Huang ^2,3*^

1 Department of General Surgery, The First Affiliated Hospital of Jinan University, Guangzhou, China

2 Department of Vascular Thyroid Surgery, The Second Affiliated Hospital of Guangzhou University of Chinese Medicine, Guangzhou, China

3 Thyroid Diagnosis and Treatment Center, Guangdong Provincial Hospital of Chinese Medicine, Guangzhou, China

4 Health Management Center, The First Affiliated Hospital of Jinan University, Guangzhou, China

5 College of Environment and Climate, Institute of Mass Spectrometry and Atmospheric Environment, Jinan University, Guangzhou, 510632, China.

#Co-first authors.

*Correspondence:

Prof. Yun-Long [Pan (tpanyl@jnu.edu.cn),](mailto:Pan(tpanyl@jnu.edu.cn),)

Prof. Xue-Yang Huang ([wkhxy@126.com](mailto:wkhxy@126.com))

Dr. Bin Hu ([bin.hu@jnu.edu.cn](mailto:bin.hu@jnu.edu.cn))

Figure S1. AUC values of three types of thyroid tissue samples.

Figure S2. Lipid identification of different ions using MS/MS experiments: a) m/z 798.5353, b) m/z 782.5690, c) m/z 780.5515, d) m/z 756.4728.

Table 1. Reproductivity of WT-ESI-MS detecting lipids from same tissue using different wooden tips

| **Lipid ions, m/z** | **RSD, %, n=6** |
| --- | --- |
| 725.5576 | 9.8 |
| 782.5675 | 8.7 |
| 808.5834 | 5.3 |
| 824.5597 | 5.6 |
| 832.5838 | 6.4 |
| 881.7567 | 7.5 |
